# Mujeres Fuertes y Corazones Saludables: adaptation of the StrongWomen —healthy hearts program for rural Latinas using an intervention mapping approach

**DOI:** 10.1186/s12889-017-4842-2

**Published:** 2017-12-28

**Authors:** Cynthia K. Perry, Jean C. McCalmont, Judy P. Ward, Hannah-Dulya K. Menelas, Christie Jackson, Jazmyne R. De Witz, Emma Solanki, Rebecca A. Seguin

**Affiliations:** 1 School of Nursing Oregon Health & Science University, 3455 SW US Veterans Hospital Rd, Portland, OR USA; 2 Division of Nutritional Sciences, Cornell University, 412 Savage Hall, Ithaca, NY USA; 3 Portland State University, SW Broadway, Portland, OR 1825 USA; 4 University of Portland, N Willamette Blvd, Portland, OR 5000 USA

**Keywords:** Health promotion, Rural, Health behavior, Latina

## Abstract

**Objective:**

To describe our use of intervention mapping as a systematic method to adapt an evidence-based physical activity and nutrition program to reflect the needs of rural Latinas.

**Methods:**

An intervention mapping process involving six steps guided the adaptation of an evidence based physical activity and nutrition program, using a community-based participatory research approach. We partnered with a community advisory board of rural Latinas throughout the adaptation process.

**Results:**

A needs assessment and logic models were used to ascertain which program was the best fit for adaptation. Once identified, we collaborated with one of the developers of the original program (StrongWomen - Healthy Hearts) during the adaptation process. First, essential theoretical methods and program elements were identified, and additional elements were added or adapted. Next, we reviewed and made changes to reflect the community and cultural context of the practical applications, intervention strategies, program curriculum, materials, and participant information. Finally, we planned for the implementation and evaluation of the adapted program, *Mujeres Fuertes y Corazones Saludables*, within the context of the rural community. A pilot study will be conducted with overweight, sedentary, middle-aged, Spanish-speaking Latinas. Outcome measures will assess change in weight, physical fitness, physical activity, and nutrition behavior.

**Conclusions:**

The intervention mapping process was feasible and provided a systematic approach to balance fit and fidelity in the adaptation of an evidence-based program. Collaboration with community members ensured that the components of the curriculum that were adapted were culturally appropriate and relevant within the local community context.

## Background

Obesity, physical inactivity, and diet are associated with increased risk of, and morbidity and mortality from, a range of chronic diseases and certain cancers [1]. In 2015, only 21% of US adults met the 2008 US Physical Activity Guidelines for both aerobic and muscle-strengthening activities [2]. Latino adults were even less likely to meet these guidelines, with only 16% meeting the guidelines compared to 23% of non-Hispanic white adults [2]. Overall, less women (18%) than men (25%) met the guidelines [2]. Rural Latinos are more likely to be obese (OR = 1.47) and inactive (OR = 1.2) than rural non-Hispanic whites [3]. The majority of Latinos do not meet the US dietary recommendations, which were developed to reduce individuals’ health risk [4]. Rural Latinos report being challenged by a traditional diet that can sometimes be unhealthy (e.g. high carbohydrates and fats with limited vegetables) and a mainstream US diet rich in processed and fast foods [5]. In 2015, there were just over 56 million Latinos in the US, which was 17% of the US population. This number is projected to increase to 119 million, 28% of the US population, by 2060 [6]. Therefore, health disparities within this population merit increased national attention.

One approach to promoting physical activity and healthy eating is the use of individually adapted behavior change programs, which are recommended in The Community Guide (www.thecommunityguide.org). In order to enhance the potential to promote change, behavior change programs need to address differing social and cultural contexts across communities. Implementing an evidence-based intervention (EBI) often involves adaptation in order to fit the local context, including cultural, socioeconomic, and rural/urban factors, to resonate with the local community and enhance effectiveness [7, 8]. Adapting an EBI to meet the needs of a local community while ensuring the core evidence-based elements remain intact poses challenges. Using a framework to guide this process can assist in striking a balance between fit and fidelity. We used a novel application of the intervention mapping process to guide our adaptation of an evidence-based physical activity and nutrition program. Intervention mapping, which has been widely used for the development of new interventions, is a systematic and iterative process that informs the development, implementation, and evaluation of health promotion interventions [9]. Applying the intervention mapping process to program adaptation involved identifying an appropriate evidence-based program that aligned with community needs and interests and adapting the identified intervention without changing its core elements [10].

The purpose of this paper is to describe our application of a method, intervention mapping to adapt an evidence-based and widely disseminated physical activity and nutrition program, StrongWomen – Healthy Hearts [11, 12], for a rural Latina population.

## Methods

### Adaptation framework

The application of intervention mapping to program adaptation consists of six steps: conducting a needs assessment; creating logic models of change and of the program; reviewing theoretical methods and adding theoretical and practical applications to match new objectives; producing the adapted program; planning program implementation; and, planning program evaluation (Table 1). We describe how we completed each of these steps within the results section of this paper. Steps 3 through 6 of this process occurred during 2015–2016. This adaptation process was approved by Oregon Health & Science University Institutional Review Board.

**Table 1:** Description of Intervention Mapping Steps for Adaptation

Step	Description
1: Needs Assessment	Assess community resources, barriers and facilitators for physical activity and healthy eating. Inform the creation of logic models, identification of EBI and adaption of EBI to local context.
2: Logic Models	Create logic model for change that delineates how to promote physical activity and healthy eating based on needs assessment. Create logic models of EBIs that most closely match community context and goals. Compare logic models of EBIs side-by-side with logic model to promote change to ascertain the EBI that needs least adaptation. Building from logic model for the identified EBI, create logic model for adapted program by adding or deleting program objectives of behavior change and specific strategies to achieve objectives.
3: Theoretical Methods	Ensure all theoretical methods and essential program elements remain. Add theoretical methods (such as family involvement) and practical applications (such as family event) for local context and population.
4: Producing the Adapted Program	Adapt structural elements, contextual elements, practical applications and specific intervention strategies so that program is responsive to the local context and population.
5: Program Implementation Planning	Plan details of implementation.
6: Evaluation Planning	Plan evaluation of program.

### Community-academic partnership and setting

We used a community-based participatory research (CBPR) approach and involved community members in the adaptation process to ensure that the evidence-based program was both sensitive and relevant to the local community. For this project, we created a community advisory board (CAB), consisting of five Latinas representing various constituencies in the community (e.g. parent of young children, high school employee, local non-profit worker). A community member who has worked with one of the researchers for many years invited Latinas living in the community to participate in the project and to be a member of the CAB. In concert with the principles of CBPR, researchers and community members shared equitably in the decision-making process and were involved in all phases of the adaptation and research process.

The study took place in a town of nearly 16,000 residents within a rural, agricultural county of Washington State with a population density of 52 people per square mile and Rural-Urban Commuting Area Code (RUCA) of 4.2, meaning a large rural area. In 2010, 82% of the town residents were Latino, 30.5% were foreign born, 73% spoke a language other than English in the home, and 49% had not earned a high school diploma [13]. The median household income was $35,699, with 25% of residents living below the poverty level [13].

## Results

We describe our application and results for each of the 6 steps in the invervention mapping.

### Step 1: Needs assessment

Four focus groups were held with Latinas in the community over the course of a year (2011–2012) to elicit their perspectives on determinants of physical activity and healthy eating and to gather intervention ideas. Convenience sampling was used to recruit participants. Latinas who participated in two informal walking groups in the community were invited to participate in a focus group after the completion of a walking group session. All Latinas in each of the two walking groups (11 women total, six in one and five in the other) agreed to participate. The trained facilitator who held the groups in Spanish took notes during each of the 45-min focus groups. A qualitative researcher identified key determinants from the notes and from a discussion with facilitator. Women whose children had participated in a research study assessing an after school physical activity program were invited to participate in a focus group. Two focus groups were held in English with eight women in one group and six in the second group. The qualitative researcher who led the groups took notes during the groups and identified key determinants from these notes.

The focus group resulted in some key findings, such as exercising in a group was a motivator to engage in physical activity. Participants were uncomfortable exercising or walking for exercise in public places, for example in parks. Additionally, participants identified involving family members in exercise and exercising as a family as important. Barriers identified included lack of time, lack of places to be active, and lack of opportunities to be active. Some stated that language was a barrier to using traditional exercise facilities. They wanted to exercise regularly; however, most did not exercise regularly and did not feel confident that they could start and maintain a routine of regular exercise. Participants identified several barriers to healthy eating, including limited access to affordable healthy foods, lack of time, and lack of knowledge in how to prepare healthy meals. Some expressed the belief that healthy foods are tasteless and that they would need to give up the foods they enjoyed in order to be healthy. They voiced interest in an exercise program that included information on healthy eating and, in particular, included cooking classes with healthy recipes.

In 2014, we conducted environmental audits, using the Nutrition Environment Measures Survey (NEMS) [14] to assess the availability of healthy foods and the Rural Activity Living Assessment (RALA) [15] to assess the community’s physical activity friendliness. The 18 convenience/corner stores that were scattered in the surrounding neighborhoods had very few healthy food options, while the three grocery stores with healthier food choices were situated along the town’s main thoroughfare. Although there are neighborhood parks and larger more centrally located parks, they were rated of poor quality. Both public and private organizations offered group exercise classes; however, neither offered low cost/sliding scale group classes [16].

### Step 2: Logic models

This step involved creating a series of logic models, a visual depiction of the strategies and activities that will lead to change [10]. From a literature search and review of databases cataloging EBI, such as National Cancer Institute’s Research Tested Intervention Programs, we identified three possible EBIs to adapt. These were StrongWomen - Healthy Hearts (SWHH) [11, 12], an exercise and nutrition program developed for rural women; Pasos Adelante [17–19], a heart health and diabetes prevention curriculum with an exercise component designed for Latinos; and, a culturally tailored aerobics intervention for low-income Latinas, which included both exercise and nutrition education (no program name) [20].

First, we created a logic model of the problem (identifying the pathway from personal determinants of the behavior and behavioral and environmental factors → the health problem). Next, logic models of change were created (identifying the pathway from program objectives designed to invoke behavioral and environmental changes → the change in behavior and the environment → the desired health outcomes) for the three identified evidence-based programs. An additional logic model was developed to delineate how to promote physical activity and healthy eating in this local community. Next, we completed a side-by-side comparison of the logic models to determine which of the three EBIs was the best fit for adaptation considering determinants addressed, behavior change strategies, culture, core elements that cannot be changed, content, delivery, level of evidence, and degree of adaptation needed.

Based on this step, SWHH was identified as the most suitable program to adapt for this community of rural Latinas. Pasos Adelante was not suitable, as some of the elements that would need to be adapted were core elements of the program. The culturally tailored aerobics intervention did not address many of the determinants for physical activity and healthy eating identified by the local community. Additionally, it had different performance objectives with the main focus of the program being 60-min classes of vigorous activity delivered three times a week for six months. In comparison, the SWHH program addressed many of the same determinants for physical activity and healthy eating (it was designed for rural white women who identified many of the same determinants as rural Latinas); it contained similar performance objectives with a focus on both moderate physical activity and healthy eating; and, its core elements (e.g. social support, enhancing knowledge) matched key elements needed for the community to change. Thus, SWHH most closely matched the community’s matrix of change and, therefore, would involve the least amount of adaptation to meet this local community’s needs. An example of matching change and performance objectives and behavioral outcomes into a matrix is depicted in Table 2. SWHH is a 12-week program of twice weekly classes. Each class lasts 60 min and consists of 30 min of moderate-vigorous physical activity (e.g. aerobics) and 30 min of hands-on preparation of healthy recipes and discussion of nutrition related topics.

**Table 2:** Select Change and Performance Objectives and Behavioral Outcomes

Change Objectives	Select Performance objectives	Behavioral outcomes
Healthy diet	Prepare healthy meals • Know what constitutes healthy diet • Purchase healthy food • Know how to cook healthy meal • Try new recipes Reduce fat & refined carb intake • Purchase low fat options • Learn how to read food labels • Purchase lean meats • Limit sweetened beverage intake • Limit sweets (muffins, cookies, cake, etc.) • Limit processed foods Consume 5 fruits & vegetables per day • Purchase fruit & vegetables • Include a fruit and/or vegetable at each meal • Eat fruits or vegetables as snacks • Know how to cook fruit and vegetables so tasty & attractive to self and family	• Prepare healthy meals • Reduce refined carbs intake • Reduce fat intake • Consume 5 fruits & vegetables per day
Sufficient physical activity for health	Know amount physical activity needed for health • Describe what type exercise, how long, how often, how hard Determine if achieving moderate-vigorous physical activity • Learn how to assess level of exertion • Practice achieving moderate-vigorous level physical activity Engage in daily moderate-vigorous physical activity for 30 min • Feel confident can fit into daily life • Feel confident on skills • Understand the connection of values to daily physical activity, • Notice change in energy level, sleep and mood • Monitor progress • Find exercise enjoyable • Not be embarrassed by skill/fitness level	• Engage in 30 min of moderate-vigorous physical activity per day

### Step 3: Theoretical methods

The developers shared the SWHH curriculum (detailed class-by-class guide) and program leader training manual. Both the CAB members and researchers reviewed the curriculum from a broad perspective looking for key determinants. Next, we identified theoretical methods and program elements or practical applications that needed to be added or adapted [10]. For example, family engagement was a program objective in the logic model for the local community and this objective is not part of the SWHH program. Thus, we incorporated this objective in the adapted program through the addition of a family celebration at the end of the 12-week program. Social support was also an important objective identified in the community logic model. While there are program elements or practical applications that encourage social support in SWHH, we determined it was important to enhance this objective. Therefore, we added an additional six weeks of informal sessions to strengthen group cohesion and support, while minimizing the need for additional resources. In reviewing the translation of the theoretical methods into practical applications we considered the conditions under which the practical applications would be effective and whether any changes were needed for the local context. For example, in order for the class leaders to serve as role models, we determined that they needed to be Latina, of similar age, and from the community so that participants could relate to and identify with them.

### Step 4: Producing the adapted program: Structural and contextual elements and intervention strategies

In this step, the researchers held a series of meetings with the CAB members to examine the class guides (curriculum), participant information sheets, recipes, and the presentation of ideas and messages (including images) and to provide feedback on necessary adaptations to align the curriculum with the local community resources, needs, and culture [10]. This feedback was recorded as comments on the curriculum and shared with the developers of SWHH through a secure cloud-based file. The developers adapted the contextual and structural elements and intervention strategies of the SWHH program materials in response to the CAB members’ recommendations. Throughout this process the researchers and the developers communicated by phone and email to clarify comments and answer questions.

The adaptations to the contextual and structural elements took the form of developing and integrating new participant information sheets (handouts), adding additional content to existing information sheets, replacing recipes, increasing exercise options, updating images, and translating all participant materials into Spanish. Each of the individual adaptations fell into at least one of the six adaptation domains: (1) Accessibility, (2) Nutrition Knowledge, (3) Health Knowledge, (4) Skills and Strategies, (5) Address Barriers, and (6) Cultural Relevance (Table 3). For example, CAB members articulated that participants might have questions about how to determine which foods are healthy. The CAB members noted that financial constraints and lack of social support are potential barriers to healthy eating and physical activity. Members of the CAB also identified foods and recipes in the SWHH program that would likely be met with resistance or disinterest. They expressed an interest in integrating healthier versions of cultural dishes and familiar exercises, such as Latin dancing, into the curriculum. Other changes to the SWHH curriculum were included to address health risks that disproportionately affect Latinas, most notably type 2 diabetes. The CAB members prepared some of the revised recipes to assess for ease of preparation, ability to obtain ingredients, and taste. Overall, they found the revised recipes acceptable. The CAB members reviewed the revised curriculum, recipes, and information sheets fully to ensure that the revisions accurately incorporated their feedback. During this review, a few minor remaining editing changes were made.

**Table 3:** Domains, goals and specific examples of adaptations made to the SWHH curriculum for rural Latinas

Adaptation Domain	Goal of Adaptation	Specific Adaptation Examples
Accessibility	Improve program accessibility and inclusiveness	• Translated all participant materials to Spanish. • Added new and updated images within participant materials. • Bilingual Community Advisory Board Members and staff of the community partner facilitated recruitment • Bilingual staff of the community partner will lead the classes and facilitate recruitment.
Nutrition Knowledge	Increase nutrition knowledge and fill potential gaps in knowledge base	Developed and integrated new participant informational sheets (handouts) on topics related to healthy foods and foods commonly found in cultural cuisine. Examples of topics include: • The five subgroups of fruits and vegetables • Nutrition information on familiar meats and cheeses • Sugar-sweetened beverages and healthy alternatives
Health Knowledge	Provide information about health risks that affect Latinas disproportionately	• Provided additional information on heart disease risk factors • Developed and integrated a handout on type 2 diabetes
Skills and Strategies	Increase skills and strategies related to making healthy choices	Provided additional or new information on the following skill-based topics: • Selecting lean protein sources • Comparing and selecting grains • Storage of fruits and vegetables • Reading and comparing nutrition labels
Address Barriers	Address potential barriers to nutrition and exercise through skill-building	Developed and integrated skill-building information to overcome potential barriers: • Healthy eating on a budget (guidance on planning, shopping and meal preparation) • Getting support from family and friends • Managing without sufficient social support
Cultural Relevance	Increase cultural relevance and appeal of the program’s recipes and exercises	• Replaced guidance on culturally unfamiliar or potentially unappealing foods with guidance on more culturally relevant foods. • Added guidance on selecting and preparing familiar food items (e.g. Tips on selecting and preparing chicken in heart-healthy ways) • Introduced new healthy recipes that use familiar ingredients • Provided recipes for healthier versions of foods commonly found in cultural cuisine • Added Latin dancing as exercise option

### Step 5: Program implementation planning

In planning for the implementation of the adapted program, *Mujeres Fuertes y Corazones Saludables,* we reviewed the training and program protocol, taking into account community resources and needs. We partnered with a local nonprofit that serves immigrant and migrant women and their families to implement the program. Sixty minute classes will be held twice weekly in the evening for 12 weeks and will be followed by six weeks of informal sessions. In the dissemination of the SWHH program, classes are led by agricultural extension agents with educational training in nutrition and physical activity; however, this was not feasible in this local community. Two local bilingual staff of the community partner, who have intimate knowledge of the local community and experience in community education, will lead the classes. They completed online training on the original SWHH curriculum and participated in a supplemental training, which focused on leadership skills, motivational interviewing and the adaptations specific to *Mujeres Fuertes y Corazones Saludables.*

A pilot study will be conducted using a one-group design. Participants will be recruited using multiple approaches, including bilingual (Spanish and English) flyers, newspaper advertisements, and radio announcements. Members of the CAB will distribute recruitment materials throughout the community (e.g. churches, schools) and attend local events to advertise the program and recruit participants. Inclusion criteria include Spanish-speaking Latinas, ages 40–70 years, inactive (exercise two days or less per week), BMI ≥ 24, and live in the local community. Individuals who meet these criteria will then be screened using the Physical Activity Readiness Questionnaire (PAR-Q), and those with a positive response will require health care provider permission to participate. Exclusion criteria include pregnancy and presence of a health condition precluding moderate to vigorous physical activity.

### Step 6: Program evaluation planning

The main outcomes for evaluating the adapted program are weight, cardiorespiratory fitness, physical activity, and dietary behavior. These will be measured at two time points: pre-program (T1) and post-program (T2). Table 4 lists the main outcome and feasibility and fidelity measures. Cardiorespiratory fitness will be measured with the 6-min walk test [21]; physical activity will be measured using an accelerometer worn for >10 h each day for 7 days to provide a valid and reliable estimate of physical activity [22]; and, dietary behavior will be assessed by the Food Intake Questionnaire [23].

**Table 4:** Study Measures

Outcome	Measure	Psychometrics or Source	Collection Times
Weight	Weight	Calibrated scale	T1, T2
Physical fitness	6-min walk test	Significant correlation with VO2 max = 0.90 [35]	
Physical activity	Accelerometer (worn for 7 days) [22]	Test-retest reliability =0.85 Significant correlation with VO2 max = 0.48 [36]	
Dietary behavior	Food Intake Questionnaire	Positive correlation with obesity for restaurant fast food (1.35), sugar sweetened beverage (1.04), full portion meal (1.34) and negative for breakfast (.92) [23]	
Fidelity and Feasibility	Participant Feedback Survey	Self-report survey about class content	Following each class, except class 24
	Leader Feedback Survey	Self-report survey about class content	Following each class
	Attendance	Attendance log	Start of each class
	Mid-program Evaluation	Self-report survey about likes/dislikes, changes to program	Class 11

Process evaluation will occur frequently and in multiple forms throughout the study. After each class, participants and leaders will complete a feedback survey to assess fidelity and feasibility of implementing the adaptations. The leader feedback examines the percentage of participants actively taking part in the exercise, class flow, and feasibility of covering added information during each class. For participants, feedback allows them to share any barriers they face in the class and to reflect on the appropriateness of the adaptations and their receptiveness towards the class curriculum. A mid-program evaluation will be given to the participants to gain further insight into their experiences with the classes. At the start of each class, attendance will be recorded. If necessary, participants will be contacted outside of class by one of the leaders to determine the reason for any reported absences or trends in tardiness. The principal investigator and program leaders will meet each week by phone to address any unanticipated challenges that arise and identify solutions. Results of this pilot study evaluation will be reported separately once completed.

## Discussion

We found it feasible to use the intervention mapping process to engage the local community and guide the adaptation of an evidence-based program aimed at obesity prevention through the promotion of physical activity and healthy eating. Intervention mapping employed in this unique way enabled the study team to identify core aspects of the curriculum that needed adaptation while retaining the essential core programmatic evidence-based elements. The adaptations were designed to enhance group cohesion and support for incorporating positive daily nutrition and physical activity based changes within participants’ lives, taking into account the cultural and geographic context. Collaboration with the CAB ensured that the aspects of the curriculum that were adapted were culturally appropriate and relevant to the local community. By collaborating with one of the developers of the original program, we were able to ensure that we maintained the core essential elements and did not alter the underlying logic of the intervention. The intervention mapping process allowed for a systematic and thorough examination of the community context and the program, and served as an effective approach for striking a balance between adaptation to the local context and fidelity to the original program.

Cultural adaptation is essential in order to reach and engage diverse cultural communities. A meta-analysis of cultural adaptation studies found that programs were most effective in their treatment implementation when they had a greater number of cultural adaptations targeted to the background and traditions of the population [24]. However, adaptations made must be relevant to the particular group characteristics, otherwise risking irrelevancy, cultural offense, and implementation failure [25]. Latino populations have been previously under-represented in health research [26], yet face a real need for tailored programs based on their high levels of inactivity. Obesity and diabetes rates among Latinos have been attributed to barriers such as language, socioeconomics, and lack of cultural relevance, which inhibit this population from utilizing pre-existing health programs [27]. By using a CBPR approach in the adaption process, the community members identified necessary cultural and linguistic adaptations in the strategies, contextual and structural elements, and materials.

While cultural adaption is critical given the diversity within the US, adaptation of EBIs beyond cultural considerations have become increasingly important. Adaptation enhances the relevance of a program, increases a program’s reach into the community and alignment with local resources, and addresses barriers and facilitators unique to the community [8]. Other dimensions of adaptation have been considered, including cognitive-information processing, affective-motivational characteristics and environmental characteristics [28], as well as program characteristics, characteristics of the individual implementers and implementing entity, planning and implementation processes, organizational leadership, and external factors [7, 29, 30]. In our adaption process we considered other dimensions (see Table 3) in addition to cultural relevance when adapting the theoretical methods, structural and contextual elements, specific behavior change strategies, and the implementation and evaluation processes.

There is an inherent tension between fidelity and adaptation, and various approaches for bridging this divide have been used. Despite ongoing debate, maintaining the fidelity of an intervention through the retention of essential core elements has been shown to produce program outcomes that are more similar to those of the original study [31]. There is currently no consensus on the most effective adaptation model in the literature. There are other models used to guide the adaptation process, such as the iterative health-promotion program life cycle [7] and the planned adaptation approach [32], as well as toolkits [33, 34]. The intervention mapping process allowed for obtaining a balance between fit and fidelity because of the detailed and systematic approach that involved assessing the community context, identifying evidence-based strategies of the original program, and adapting to local context while retaining evidence-based strategies.

## Conclusions

Our use of intervention mapping as a systematic approach for the adaptation of an EBI provided a guide for a deep dive into the community context and a comprehensive examination of all aspects of the EBI, including implementation and evaluation. This use of intervention mapping contributes to the growing body of research on intervention adaptation by demonstrating that intervention mapping can be re-appropriated and effectively applied to the intervention adaptation process using a community-engaged approach within a community-based setting.

## Abbreviation

EBI: Evidence-based intervention
